# Tangential beam IMRT versus tangential beam 3D-CRT of the chest wall in postmastectomy breast cancer patients: A dosimetric comparison

**DOI:** 10.1186/1748-717X-6-26

**Published:** 2011-03-21

**Authors:** Volker Rudat, Abdul Aziz Alaradi, Adel Mohamed, Khaled AI-Yahya, Saleh Altuwaijri

**Affiliations:** 1 Department of Radiation Oncology, Saad Specialist Hospital, P.O. Box 30353, Al Khobar 31952, Saudi Arabia; 2 SAAD Research & Development Center, Saad Specialist Hospital, P.O. Box 30353, Al Khobar 31952, Saudi Arabia

## Abstract

**Background:**

This study evaluates the dose distribution of reversed planned tangential beam intensity modulated radiotherapy (IMRT) compared to standard wedged tangential beam three-dimensionally planned conformal radiotherapy (3D-CRT) of the chest wall in unselected postmastectomy breast cancer patients

**Methods:**

For 20 unselected subsequent postmastectomy breast cancer patients tangential beam IMRT and tangential beam 3D-CRT plans were generated for the radiotherapy of the chest wall. The prescribed dose was 50 Gy in 25 fractions. Dose-volume histograms were evaluated for the PTV and organs at risk. Parameters of the dose distribution were compared using the Wilcoxon matched pairs test.

**Results:**

Tangential beam IMRT statistically significantly reduced the ipsilateral mean lung dose by an average of 21% (1129 cGy versus 1437 cGy). In all patients treated on the left side, the heart volume encompassed by the 70% isodose line (V70%; 35 Gy) was reduced by an average of 43% (5.7% versus 10.6%), and the mean heart dose by an average of 20% (704 cGy versus 877 cGy). The PTV showed a significantly better conformity index with IMRT; the homogeneity index was not significantly different.

**Conclusions:**

Tangential beam IMRT significantly reduced the dose-volume of the ipsilateral lung and heart in unselected postmastectomy breast cancer patients.

## Background

Breast cancer is the most common cancer in females worldwide. In the United States and Europe, the most common treatment is breast conserving surgery followed by adjuvant radiotherapy [1]. In other parts of the world including the Middle East, the majority of the patients present in a more advanced stage of disease at diagnosis, and mastectomy is the most common treatment followed by adjuvant radiotherapy of the chest wall [2].

Large prospective trials [3] and a meta-analysis [4] have shown that adjuvant radiotherapy of the chest wall improves local control and survival in node positive breast cancer patients after mastectomy. The adjuvant radiotherapy of the chest wall is commonly achieved with tangential beams, similar to the treatment technique used for the adjuvant whole breast radiation in early breast cancer. The tangential beams include part of the anterior thoracic cavity, thereby potentially affecting the organs at risk, in particular the lung and heart.

Randomized, retrospective and population based studies have shown that the radiotherapy of the chest wall is associated with a significantly increased risk of developing ipsilateral second lung cancer [5-12], and in patients treated on the left side with a significantly increased risk of cardiac morbidity and mortality [4,13-24].

There is a good body of literature showing that inversed planned intensity modulated radiotherapy (IMRT) potentially leads to a more favourite dose distribution compared to three-dimensional planned conformal radiotherapy (3D-CRT) for the radiotherapy of the whole breast after breast conserving surgery [25-48]. Data on the effect of IMRT of the chest wall in post-mastectomy breast cancer patients are scarce in the literature [49-51]. There are distinct differences between the target volume of the chest wall and the whole breast. The shape of the target volume of the chest wall is usually shallower compared to the whole breast. In addition, in stage I-IIa patients the pectoralis muscle, chest wall muscles, and ribs may be excluded in the target volume of the whole breast, whereas these structures are included in the target volume of the chest wall. Due to these differences in the target volume, results of a dosimetric study of the radiotherapy of the whole breast may not be completely applicable to the radiotherapy of the chest wall.

This study specifically evaluates the dose distribution of tangential beam IMRT of the chest wall in postmastectomy breast cancer patients compared to tangential beam 3D-CRT.

## Methods

### Patient data

For 20 unselected consecutive postmastectomy breast cancer patients an opposed tangential beam IMRT plan and a standard opposed tangential beam 3D-CRT plan was generated for the radiotherapy of the chest wall. Thirteen patients had right-sided breast cancer and seven left-sided. The target volumes were defined and the dose prescribed according to the International Commission on Radiation Units and Measurement (ICRU) Reports 50 and 62 recommendations. Accordingly, the target volume should be surrounded by the 95% isodose line. The planning target volume (PTV) definition for the chest wall was done according to the breast cancer atlas for radiation therapy planning consensus definitions of the Radiation Therapy Oncology Group (RTOG) http://www.rtog.org/CoreLab/ContouringAtlases/BreastCancerAtlas.aspx. The PTV included the chest wall with the pectoralis muscle, chest wall muscles, and ribs, and excluded the outermost 3 mm from the superficial skin surface. The heart was defined as all visible myocardium, from the apex to the right auricle, atrium, and infundibulum of the ventricle. The pulmonary trunk, root of the ascending aorta, and superior vena cava were excluded.

This retrospective planning study was approved by the Institutional Review Board and Ethics committee. For the statistical analysis, the patient data were anonymized to guarantee privacy.

### Treatment techniques

A non-contrast CT-simulation was performed in the supine position on a carbon breast board with the ipsilateral arm up and head turned to the contralateral side. Radio-opaque wires were used to mark the mastectomy scar and the clinical boundaries. A CT scan was performed using 5 mm slice thickness. The CT scanning reference point was defined using the CT simulation software Coherence Dosimetrist (Siemens Medical), and target volumes (PTV and organs at risk) using the software Coherence Oncologist (Siemens Medical). The 3D-CRT and IMRT plans were generated using the treatment planning system XIO 4.4 (CMS, Inc. of St. Louis, Mo, USA). A Siemens Oncor Anvantgarde linear accelerator with dual photon energy of 6 MV and 15 MV and multileaf collimator was used for the treatment. The leaf width was 1 cm at the isocenter. The dose calculation was determined using the "Superposition" algorithm. The prescribed total dose was 50 Gy in 25 fractions. The beam energy of 6 MV was used for all 3D-CRT and IMRT plans because of the better dose coverage of the chest wall due the lower penetration power compared to 15 MV.

#### Tangential beam 3D-CRT

The dose was prescribed to the ICRU reference point which was usually the isocenter located in the PTV volume centroid. Two tangential semi-opposed beams (to avoid divergence), physical wedges (usually 15° or 30°), and a multileaf collimator were used for 3D-CRT. The beam angles, wedge angles, and beam weighting (usually minimal) were chosen to optimize coverage of the PTV, while minimizing exposure to the ipsilateral lung, heart and contralateral breast. Gantry angles ranged from 42° to 55° for the medial fields and from 224° to 232° for the lateral fields for patients treated on the right side, and from 305° to 322° for the medial fields and from 133° to 147° for the lateral fields for patients treated on the left side. The fields extended 2 cm anteriorly of the chest to provide coverage of the "flash" region.

#### IMRT technique

The same beam orientations and angles of the 3D-CRT plan were used for the tangential beams of the corresponding IMRT plan. The PTV included the same PTV used for the 3D-CRT plans plus an extension into the air anteriorly of the chest of 1.5 cm to ensure appropriate opening of the multileaf collimator. The dose was prescribed to the PTV, and as initial dose volume constraints the IMRT prescription table provided by the XIO treatment planning system was used (Table 1). Tissue inhomogeneities were considered in the treatment planning optimization process, and the dose calculation algorithm used was "Superposition". A step-and-shoot technique was applied. An optimization with 100 iterations was then applied, and followed by a semiautomatic segmentation (minimum 3 cm step size). Segments with less than ≤2 MU were expelled from the plan.

**Table 1:** Dose-volume constraints for IMRT plans.

Structure	Type	Rank	Objective	Dose (cGy)	Volume (%)	Weight
PTV	Target	1	Maximum	5200	0	100
PTV	Target	1	Minimum	4900	100	100
Ipsilateral lung	Organ at risk	2	Maximum	2000	20	100
Ipsilateral lung	Organ at risk	2	Minimum	1200	30	100
Heart	Organ at risk	3	Maximum	4500	0	100
Unspecified tissue	Organ at risk	4	Maximum	4500	0	100

IMRT, intensity modulated radiotherapy; PTV, planning target volume.

Dose volume histograms of the PTV and organs at risk of the 3D-CRT and IMRT plans were generated and dose parameters compared. The Homogeneity index (HI) was defined as the fraction of the PTV with a dose between 95% and 105% of the prescribed dose (V_95% _- V_105%_). The Conformity Index (CI) was defined as the fraction of the PTV surrounded by the reference dose (V95%) multiplied by the fraction of the total body volume covered by the reference PTV dose ((PTV_95% _/PTV) × (PTV_95% _/V_95%_)).

### Statistics

IMRT and 3D-CRT plan parameters derived from the same patient were tested for statistically significant difference using the Wilcoxon matched pairs test. All *P *values were two-tailed. No correction for multiple testing was used.

## Results

**Figure 1:**
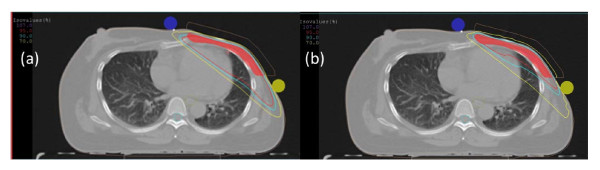
**Dose distribution (V107%, V95%, V90%, V70%) for (a) conformal three-dimensional (3D-CRT) and (b) intensity modulated radiotherapy (IMRT) plans**.

Table 2 compares plan parameters of opposed tangential beam IMRT with conventional 3D-CRT for the adjuvant radiotherapy of the chest wall in 20 unselected consecutive breast cancer patients after mastectomy. Figure 1 demonstrates typical dose distributions of an IMRT and 3D-CRT plan of the same patient.

**Table 2:** Relevant plan parameters of tangential beam IMRT versus tangential beam 3D-CRT of the adjuvant radiotherapy of the chest wall in unselected postmastectomy breast cancer patients.

	IMRT		3D-CRT				
				
Organ Parameter	Mean	1SD	Mean	1SD	Difference	Difference (%)	*P *value
Ipsilateral chest wall (PTV)							
Maximum Dose (cGy)	5530	146	5462	135	68	1	0.04
Mean Dose (cGy)	5083	73	5038	70	44	1	0.04
Homogeneity Index	0.73	0.15	0.77	0.11	-0.05	-6	n. s.
Conformity Index	0.32	0.04	0.25	0.14	0.07	26	0.03
Heart*							
Maximum Dose (cGy)	3874	1729	4990	180	-1116	-22	n. s.
Mean Dose (cGy)	704	295	877	272	-173	-20	0.03
V70%	5.71	3.40	10.61	3.68	-4.90	-46	<0.03
Ipsilateral lung							
Mean Dose (cGy)	1129	188	1437	204	-308	-21	<0.01
D30%	960	537	1695	875	-734	-43	<0.01

3D-CRT, three-dimensional conformal radiotherapy; IMRT, intensity-modulated radiotherapy; 1SD, standard deviation; V70%, percentage of tissue volume encompassed by the 70% isodose line (35 Gy); D30%, dose to 30% of the volume (PTV or Organs at risk); *, Patients with left-sided breast cancer only; n.s., not significant.

Concerning the PTV (chest wall), tangential beam IMRT significantly improved the conformity index compared to 3D-CRT. The maximum and mean dose was higher in the IMRT plans, but the differences were small (about 1%). The Homogeneity Index was not significantly different between the IMRT and 3D-CRT plans.

All patients treated on the left side showed a reduction of the V70% (percentage of volume encompassed by the 70% isodose line; corresponding to the volume receiving ≥35 Gy) of the heart with an average of 43% (*P *< 0.01). The mean heart dose was reduced by an average of 20%. The ipsilateral mean lung dose was statistically significantly reduced by an average of 21%.

The mean volume and the standard deviation (1SD) of the PTV (chest wall) was 612.0 cm^3 ^(173.7 cm^3^), of the heart 524.2 cm^3 ^(125.5 cm^3^), and of the ipsilateral lung 1136.7 cm^3 ^(244.4 cm^3^).

## Discussion

A number of studies have demonstrated a dosimetric benefit of IMRT compared to 3D-CRT for the whole breast in early breast cancer patients. Data about the impact of IMRT on the adjuvant radiotherapy of the chest wall in postmastectomy patients are scarce in the literature. There are distinct geometric differences between the target volume of the chest wall and the whole breast, and these differences might have an impact on the resulting dose distribution. This study was undertaken to evaluate the dose distribution of tangential beam IMRT of the chest wall compared to tangential beam 3D-CRT in unselected postmastectomy breast cancer patients.

Our data show that tangential beam IMRT of the chest wall compared to 3D-CRT significantly reduces the ipsilateral lung dose-volume (D30% by 43%), and heart dose-volume in patients treated on the left side (V70% by 46%). Similar results have been reported for tangential beam IMRT for the whole breast in early breast cancer patients. In a recent study, Smith et al. compared three tangential beam IMRT plans with conventional tangential beam 2 D plans for the adjuvant radiotherapy of the whole breast in 20 patients with early breast cancer [52]. All IMRT plans showed a significant improvement of the PTV homogeneity index of 15%, heart V30% of 28-33%, and whole lung V20% of 2-8% compared to the conventional technique.

A significantly better sparing of the high-dose volume of the heart in selected early breast cancer patients with unfavourable thoracic geometry has been reported by the use of multifield IMRT [53,54]. Compared to 3D-CRT, multifield IMRT reduced the heart volume receiving ≥30 Gy by 87% [53], or ≥35 Gy by 81% [54]. Model calculation using a relative seriality model [55] suggested that the excess cardiac risk was decreased from approximately 6% to <1% in these patients [53]. On the other hand, in contrast to our study using tangential beam IMRT, multifield IMRT significantly increased the mean heart dose by an average of 24.4% [53], the left lung D30% by 143% [53], and the volume of the left lung receiving ≥20 Gy by 47%[54].

It is difficult to precisely estimate the possible clinical effect of the heart dose-volume reduction by the use of multifield versus tangential beam IMRT. Clinically recognized presentations of radiation induced heart disease have been observed in patients who received therapeutic doses of about ≥35 Gy to partial volumes of the heart [56]. Recent studies based on atom bomb survivors also suggest a relationship between cardiac mortality and low radiation doses in the range of ≤4 Gy [57-60]. The development of radiation-related heart disease is a complex process involving different heart structures with different radiosensitivities and pathomechanisms, and is still not well understood [61,62]. Furthermore, pre-existing cardiovascular risk factors as smoking, obesity, and hypertension as well as the use of cardiotoxic agents such as anthracyclines, paclitaxel and trastuzumab are likely to contribute to the development of radiation-related heart disease. In view of the potential risks it has been recommended that all measures should be attempted to reduce cardiac radiation exposure [61].

An increased risk of secondary tumors has been observed in breast cancer patients treated with older radiation techniques, which combined higher radiation dose and larger tissue volumes [5,11,12,63,64]. Modern radiotherapy techniques as 3D-CRT are likely to reduce the secondary cancer risk by reducing the lung dose-volume [65]. Smoking has been shown to significantly increase the risk of second lung cancer in radiotherapy patients even if modern radiation techniques were used [66,67].

Multifield IMRT has been discussed to possibly increase the risk of second cancers [68]. The reason for this is that compared to 3D-CRT a larger volume of healthy tissue is being irradiated with lower doses due to the use of multiple beams and the high number of monitor units.

Prospective studies with long follow-up times are needed to fully evaluate the cardiac toxicity and secondary lung cancer risk in breast cancer patients treated with tangential beam or multifield IMRT.

## Conclusions

Tangential beam IMRT for the radiotherapy of the chest wall of postmastectomy breast cancer patients offers the potential to significantly reduce the dose-volume of the ipsilateral lung, and in patients with left-sided cancer the dose-volume of the heart compared to tangential beam 3D-CRT. These results are similar to those reported for tangential beam IMRT of the whole breast in early breast cancer. In selected patients with unfavourable thoracic geometry, multifield IMRT has been shown to reduce the heart high dose-volume more effectively, but on the cost of an increased mean heart dose and ipsilateral lung dose compared to tangential beam IMRT.

## Abbreviations

DX%: Dose to X% of the volume (PTV or Organs at risk); IMRT: Reversed planned intensity modulated radiotherapy; PTV: Planning target volume; VX%: Percentage of tissue encompassed by the X% isodose line, representing the volume of tissue that receives at least 95% of the prescribed dose; 3D-CRT: Three-dimensionally planned conformal radiotherapy.

## Competing interests

The authors declare that they have no competing interests.

## Authors' contributions

AA, AM, and KA participated in the study design, carried out the dose calculation, and helped to draft the manuscript. SA participated in its design and coordination and helped to draft the manuscript. VR conceived of the study, participated in its design and coordination, participated in the treatment panning, performed the statistical analysis, and drafted the manuscript. All authors read and approved the final manuscript.
